# An Implementation Strategy to Expand Mobile Health Use in HIV Care Settings: Rapid Evaluation Study Using the Consolidated Framework for Implementation Research

**DOI:** 10.2196/19163

**Published:** 2021-04-28

**Authors:** Wendy F Cohn, Chelsea E Canan, Sarah Knight, Ava Lena Waldman, Rebecca Dillingham, Karen Ingersoll, Julie Schexnayder, Tabor E Flickinger

**Affiliations:** 1 Department of Public Health Sciences University of Virginia Cancer Center University of Virginia Charlottesville, VA United States; 2 Division of Infectious Diseases Department of Medicine University of Virginia Charlottesville, VA United States; 3 Department of Psychiatry and Neurobehavioral Sciences University of Virginia Charlottesville, VA United States; 4 Division of General, Geriatric, Palliative and Hospital Medicine Department of Medicine University of Virginia Charlottesville, VA United States

**Keywords:** mHealth, smartphone, mobile health, implementation strategy, implementation science, Consolidated Framework for Implementation Research, HIV care engagement, viral suppression

## Abstract

**Background:**

Mobile health (mHealth) apps can provide support to people living with a chronic disease by offering resources for communication, self-management, and social support. PositiveLinks (PL) is a clinic-deployed mHealth app designed to improve the health of people with HIV. In a pilot study, PL users experienced considerable improvements in care engagement and viral load suppression. To promote its expansion to other HIV clinics, we developed an implementation strategy consisting of training resources and on-demand program support.

**Objective:**

The objective of our study was to conduct an interim analysis of the barriers and facilitators to PL implementation at early adopting sites to guide optimization of our implementation strategy.

**Methods:**

Semistructured interviews with stakeholders at PL expansion sites were conducted. Analysis of interviews identified facilitators and barriers that were mapped to 22 constructs of the Consolidated Framework for Implementation Research (CFIR). The purpose of the analysis was to identify the facilitators and barriers to PL implementation in order to adapt the PL implementation strategy. Four Ryan White HIV clinics were included. Interviews were conducted with one health care provider, two clinic managers, and five individuals who coordinated site PL activities.

**Results:**

Ten common facilitators and eight common barriers were identified. Facilitators to PL implementation included PL’s fit with patient and clinic needs, PL training resources, and sites’ early engagement with their information technology personnel. Most barriers were specific to mHealth, including access to Wi-Fi networks, maintaining patient smartphone access, patient privacy concerns, and lack of clarity on how to obtain approvals for mHealth use.

**Conclusions:**

The CFIR is a useful framework for evaluating mHealth interventions. Although PL training resources were viewed favorably, we identified important barriers to PL implementation in a sample of Ryan White clinics. This enabled our team to expand guidance on identifying information technology stakeholders and procuring and managing mobile resources. Ongoing evaluation results continue to inform improvements to the PL implementation strategy, facilitating PL access for future expansion sites.

## Introduction

People living with HIV achieve positive health outcomes more effectively when they establish and maintain primary HIV care and have high adherence to their antiretroviral therapy [1-3]. Mobile health (mHealth) apps can support chronic disease self-management in this population by providing a platform for communication, self-monitoring, and social support. Improved outcomes have been reported for mHealth users with chronic diseases including asthma, diabetes, and HIV [4-8]. Because of their potential for improving health outcomes, health systems are beginning to leverage mHealth interventions to engage people with HIV in self-management behaviors [9]. Accordingly, there is an unmet need for implementation strategies to support bidirectional mHealth use by people living with HIV and their primary care teams.

**Figure 1:**
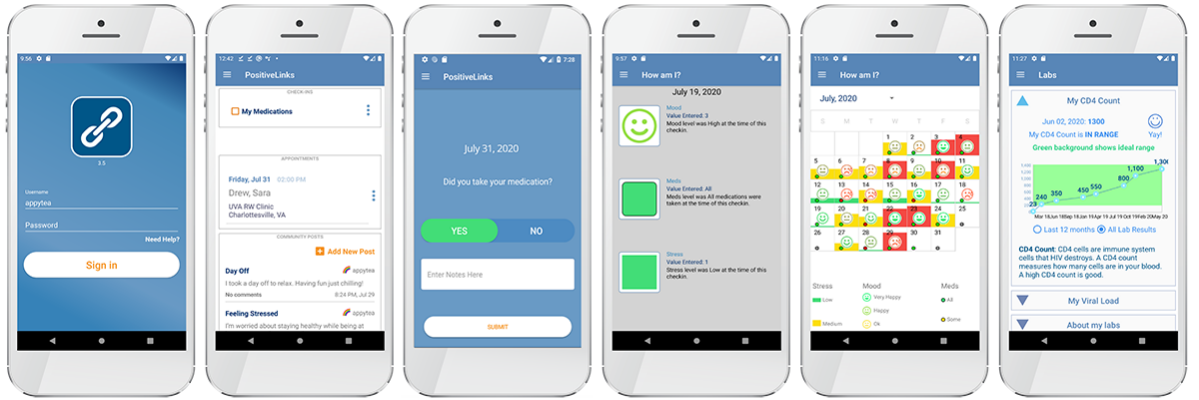
Positive Links screen shots: patient app.

*PositiveLinks* (PL) is a clinic-based mHealth intervention that was designed in partnership with people living with HIV to increase engagement in care and improve clinical outcomes [10]. PL consists of a smartphone app for patients that includes features, such as appointment reminders, a virtual community board, and daily queries of mood, stress, and medication adherence, with graphical feedback of behavioral patterns (Figure 1) paired with a suite of tools for clinics and providers. Following app development, PL was piloted at the University of Virginia’s Ryan White Clinic in a single arm prospective study. We found considerable improvements in engagement in care and in key clinical laboratory markers (CD4 count and HIV viral suppression), measures of immune system recovery, and cessation of HIV viral replication. The app was subsequently adopted as usual care at this site [11].

PL is administered by the HIV primary care clinic. At the University of Virginia, PL is coordinated by a dedicated team member who enrolls patients and HIV care team members in the program, issues smartphones and payments for smartphone services to patients, provides patient training on use of the app’s features, manages app content, and assists with phone and app troubleshooting. Selected clinical laboratory results and other medical information are directly imported from the electronic medical record to PL for patient viewing within the app. Referrals to the program are made by clinical and nonclinical members of the HIV care team. HIV care team members access PL as needed to exchange messages with patients in a password-protected environment and, if desired by team members, to monitor their patients’ self-reported medication adherence. Patients are asked to complete daily check-ins on their HIV medication use, moods, and stress, and have unrestricted access to all app features.

In 2017, PL was made available to other HIV service providers. The primary targets for PL expansion were HIV clinics that were funded under the Health Resources and Services Administration’s Ryan White HIV/AIDS Program. The objectives of this study were to describe an interim rapid evaluation of PL implementation determinants at early adopting Ryan White clinics using the Consolidated Framework for Implementation Research (CFIR) and to describe the process for refining our PL implementation support program based on the study findings.

## Methods

### Setting

The University of Virginia Institutional Review Board approved this study. The setting for this study was four Ryan White clinics that pursued PL implementation in 2018. Ryan White clinics provide HIV primary medical care, medications, and essential support services for people living with HIV who are low income, uninsured, and underserved [12]. The clinics implementing PL varied in their location and organizational structure. They included three health system-affiliated Ryan White clinics in Virginia and one in Texas. All four clinics were located in cities, with the two largest clinics serving primarily an urban population and the two remaining clinics reaching a catchment area that included both urban and rural regions. At the time of this evaluation, of the four sites, one ceased participation in PL before enrolling patients due to difficulties with leadership buy-in, one was unable to obtain information security approvals for PL use within the parent health system, and two progressed to the implementation stage and began enrolling patients.

All sites received PL implementation support from the University of Virginia PL implementation team that incorporated evidence-based interventions from an implementation research taxonomy [13]. Implementation support included a comprehensive training package consisting of a training manual, onsite PL training for HIV care team members, a PL learning management system accessible directly from PL’s clinic/provider tool suite, and ongoing and on-demand program support from an experienced PL coordinator.

We anticipated that successful implementation of PL would require processes and infrastructure on multiple levels. At the individual level, PL members (individuals living with HIV) must download and install the app and interact with the app for self-monitoring. At the interpersonal level, PL members communicate with the implementation site–specific PL coordinator and their care team. PL coordinators at the sites also interact with the University of Virginia PL implementation team for training and support. PL workflow must be integrated into the clinic site and processes developed for allocation of phone resources, member enrollment, and support and engagement for members and providers. At the organizational level, the PL program requires leadership approval and information technology (IT) infrastructure to support it.

### Recruitment and Study Participants

**Figure 2:**
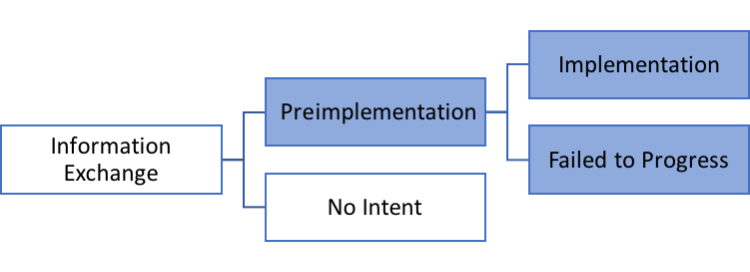
Stages of implementation. Interviews occurred during the stages shaded in blue.

Purposive sampling was used to recruit employees from the Ryan White clinics that expressed intent to adopt the PL program. Intent to adopt PL was ascertained from direct email inquiries, personal contact with the investigators, or requests for information on the PL website. The PL implementation coordinator at the University of Virginia tracked these clinics in their progression toward PL implementation. Figure 2 displays the stages of the implementation pathways from *information exchange* when potential sites contact PL to learn about the program to *preimplementation* after the decision is made to implement PL and then to either *implementation* or *failed to progress*. Sites were included in the evaluation when the clinic made the decision to adopt PL (preimplementation). The evaluation team was notified to initiate interview recruitment activities and received contact information for the individual confirming intent to implement PL. This individual was contacted by phone or email and asked to identify primary stakeholders involved with the PL implementation process at the site. The individuals were then approached to participate in the study via email.

We specifically targeted clinic managers, PL coordinators, and PL providers for interviews. PL coordinators were the point people for PL at each site; they were responsible for enrollment and support of PL members. We defined PL providers as physicians, nurses, psychologists, social workers, case managers, community health workers, or any other staff members who were end-users of the PL platform.

### Implementation Framework Selection

An implementation science determinants framework informed the interview guide development and analysis, and it allowed for consideration of the multilevel factors that may contribute to implementation success [14,15]. The CFIR [16] captures a broad range of constructs that fit the goals of this evaluation and provides interview guides and codebook templates that facilitate the application of this framework to evaluation projects [17]. At the time of our evaluation, the CFIR was being used widely in health-related implementation research [18-27] but had a smaller presence in the field of mHealth [28-31].

The CFIR consists of the following five domains: *intervention characteristics*, *outer setting*, *inner setting*, *characteristics of individuals*, and *implementation process*. Across the primary domains are 39 specific constructs corresponding to factors of successful implementation [16,17]. The CFIR is designed to be flexible in its use, with users able to determine which constructs are most relevant to their project’s implementation.

Two sources guided the selection of specific CFIR constructs for inclusion in the evaluation. The University of Virginia PL implementation team reviewed and identified all five domains and six priority constructs (innovation source, evidence strength and quality, patient needs and resources, self-efficacy, and engaging opinion leaders) as most relevant to the PL implementation based on their experiences with early adopting sites. Additionally, a 2016 systematic review summarized factors that influence the implementation of all types of eHealth interventions [31]. We used findings from the systematic review to select CFIR constructs most relevant to mHealth interventions. Unlike mHealth interventions described in the report by Ross et al [31], cost was not anticipated to be a barrier to initial PL implementation. All four Ryan White clinics had received grant funding to support their PL programs. In total, 22 CFIR constructs were included in the analysis. The chosen constructs with their PL-specific operational definitions are included in Table 1. We also included an additional code for *technology* because the mHealth aspect of the intervention was particularly salient to our implementation, and we wanted to be sure that there was a code to quickly capture all data related to this theme.

### Interview Guide Development

We developed three interview guides to permit tailoring of questions to clinic managers, PL coordinators, and PL providers. PL coordinators were asked questions about the PL program at two time points (before implementation and during implementation) using the same interview guide. PL providers were asked questions about the PL program during implementation only (ie, after the clinic began enrolling patients in the PL program). Interview guides incorporated questions associated with 22 CFIR constructs (Table 1). For each construct, we began by adapting questions suggested by the CFIR developers [17] and added questions as necessary for our particular project needs.

**Table 1:** Included Consolidated Framework for Implementation Research constructs and their operationalization.

Construct		Before implementation	During implementation	
		Coordinator	Coordinator	Provider
**Innovation characteristics**				
	Innovation source “*Who developed PL*^a^*? Why is PL being implemented in your clinic?”*	No	No	Yes
	Evidence strength and quality “*What evidence are you aware of that shows whether PL will work in your clinic?”*	No	No	Yes
	Adaptability “*What changes will you need to make to PL so it works in your clinic?”*	Yes	No	No
	Complexity “*How complicated is PL?”*	No	No	Yes
	Design quality and packaging “*What is your perception of supporting materials, packaging and bundling of PL?”*	No	Yes	Yes
**Outer setting**				
	Patient needs and resources *"How well does PL meet the needs of your patients? How do patients respond to PL?”*	No	Yes	Yes
	External policy and incentives *"What local, state, national policies or guidelines influenced your decision to implement PL?”*	Yes	No	No
**Inner setting**				
	Compatibility “*How does PL fit with the values and norms in your clinic? How does PL fit into clinic processes and workflow?”*	Yes	No	Yes
	Leadership engagement “*What kind of support for PL have you seen from leaders in your clinic?”*	Yes	Yes	No
	Available resources “*What resources do you need to implement PL? Do you have those resources?”*	Yes	No	Yes
	Access to knowledge and information “*What kind of training is planned for you and your colleagues?”*	Yes	No	Yes
**Characteristics of individuals**				
	Knowledge and beliefs about the intervention “*Do you think PL will be effective? How do you feel about your plan to implement PL?”*	Yes	No	Yes
	Self-efficacy “*How confident do you feel about implementing PL?”*	Yes	No	Yes
	Other personal attributes “*Tell me about yourself and your role with PL”*	Yes	No	Yes
**Implementation process**				
	Planning “*To what extent is there a plan in place to implement PL? Who is involved? What role has your plan played in implementation?”*	Yes	Yes	No
	Engaging opinion leaders “*Who are key individuals to get on board? What are they saying about PL?”*	Yes	Yes	No
	Engaging formally appointed internal implementation leaders “*Who will lead PL implementation? How did your clinic get involved in PL?”*	Yes	No	No
	Engaging champions “*Are there people who go above and beyond what might be expected?”*	Yes	No	No
	Engaging key stakeholders “*What steps are taken to encourage participation in PL?”*	No	No	Yes
	Engaging innovation participants “*How do you communicate PL to patients?”*	No	No	Yes
	Executing “*Has PL been implemented according to plan?”*	No	Yes	Yes
	Reflecting and evaluating “*What kind of information do you collect as you implement PL? How do you assess progress toward your goals?”*	Yes	Yes	No

^a^PL: PositiveLinks.

### Interviews

Recruitment began at all clinics on February 15, 2018. Interviews were completed between March 1, 2018, and July 10, 2019. All participants verbally provided their informed consent to participate and were allowed to discontinue participation at any time after giving consent. Interviews were conducted over the phone and were audio recorded and professionally transcribed. Interview lengths differed based on the guide used, ranging from 30 to 60 minutes. Interviewers were trained members of the evaluation team, and they were not involved with PL implementation at the clinics. This was done to promote candid and honest responses from the interview participants when asked to describe their experiences. Interviews were stored as audio files on a secure drive labeled by study ID number. Individual interviews were not discussed with the entire team to preserve confidentiality.

Eight interviews were completed, including three interviews that occurred during the preimplementation stage and five interviews that occurred after the clinics began enrolling patients in their PL programs. Six of the interviews were contributed by two clinics that successfully implemented PL during the study period. The remaining clinics were unable to implement PL as of June 30, 2019. Each of these clinics contributed a single interview. One of the clinics experienced difficulties in garnering leadership buy-in for PL use. The other clinic was unable to obtain information security approvals for PL use within the parent health system.

Because only two sites progressed to enrolling patients in PL, PL providers were only eligible to participate in interviews at two sites. Of 13 providers we attempted to recruit via email, two declined interviews, one was lost to follow-up, nine did not respond, and one successfully completed the interview.

### Analysis

An analytical template was used by trained personnel (WC, CC, JS, and TEF) using the CFIR codebook [17], which provided operational definitions for CFIR constructs along with example inclusion and exclusion text. We restricted the codebook to those constructs selected for inclusion as described above, with the addition of the *technology* code as previously described. Transcripts were independently coded by two investigators who then worked together to achieve consensus on the coded content. Coding and analyses were performed using Dedoose Version 8.2.14 [32].

After reaching consensus on the coded content, the two coders independently summarized the barriers and facilitators that emerged from each interview. These summaries also underwent consensus discussions. The summaries were used to generate a master list of PL implementation determinants. Each determinant was listed with its corresponding CFIR construct–specific examples from the interviews and potential action items to report to the implementation team. Facilitators were evaluated by the team to assess whether there were corresponding actions that would enhance or strengthen the facilitator and enable future clinics to benefit from explicit recommended actions.

The master list of PL implementation determinants, including facilitators and barriers, was updated and shared with the implementation team as each determinant summary was completed. This rapid evaluation process enabled iterative changes to the implementation model prior to study completion. The median time from interview completion to determinant summary completion was 131 days and ranged from 36 to 228 days. All information presented to the implementation team was delivered in a deidentified and aggregated manner.

## Results

### Facilitators

Based on our analyses, nine CFIR constructs were associated with 10 facilitators. A summary of facilitators and recommended action items is shown in Table 2. PL *compatibility* was an important facilitator to its implementation. Components of fit included alignment between (1) PL and clinic needs, and (2) PL and clinic goals and values. The intersection between *patient needs and resources* and *compatibility* constructs was notable. For example, the respondents mentioned that accessing the clinic was a challenge for some of their patients. Having a phone and connectivity to the clinic through PL helped to address this barrier. The following two action items arose from findings related to PL compatibility: (1) remind sites that it is important to budget for cell phones for their most at risk patients and (2) help sites identify their own needs and articulate how PL addresses those needs during preimplementation.

Engaging key stakeholders was also identified as an important facilitator of PL implementation. First, stakeholder engagement with PL implementation activities contributed to leadership and end-user excitement for the program. Second, stakeholder input during implementation planning prepared teams to integrate PL into their existing workflows. Encouraging sites to include clinic staff in implementation planning was identified as an action item.

The *planning* and *engaging innovation participants* constructs identified useful strategies for boosting engagement by PL providers and users. Action items included proactive planning for these engagement strategies and timing marketing of PL features that are dependent on group participation. The remaining CFIR constructs (and their associated facilitators) highlighted aspects of the implementation process that were going well and did not require additional action. Examples include accessibility and quality of PL training materials, ease of PL use, and PL’s ability to be adapted to clinic needs. For these facilitators, the evaluation team recommended that the implementation team continue current practices.

**Table 2:** Facilitators to PositiveLinks implementation.

CFIR^a^ domain and construct: facilitator		Example	Action item
**Outer setting**			
	Needs and resources of those served: Perceived match between needs of clients and PL^b^ features	PL perceived as meeting patient needs for engagement, communication, support, medication adherence, appointments, and lab tracking Staff believe PL can help patients who have difficulty getting to the clinic Phones help patients stay in touch with the clinic and family/friends	Remind sites of the importance of budgeting for cell phones for most at risk patients Help sites identify their own needs and then discuss how PL can address these needs
**Inner setting**			
	Compatibility: PL meets the needs of the clinic/staff	PL is supporting what staff are already doing to engage patients in care Incorporating PL into clinic operations and quality management plans PL perceived as helping to overcome communication-related gaps in engagement	Emphasize that PL might make it easier for staff to do what they are already doing Remind sites that this tool was developed to meet the needs identified by clinicians
	Compatibility: PL alignment with clinic goals and values	Good alignment between goals of the clinic and PL: connecting to clients, medication adherence, and patient-centered focus	Ask clinics to identify their values and goals, for example, setting targets for retention-in-care or viral suppression rates that may be improved by PL use.
	Access to knowledge and information: Quality of PL training materials	Positive impression of training, materials, and support for both learning the program and navigating through the approval process Plan for training is well developed, occurs at an appropriate time, and is delivered to the right staff	No action indicated
**Innovation characteristics**			
	Complexity: Ease of PL use	Simplicity and user friendliness of the patient-facing app Web portal viewed as simple and easy; made it easier for staff to use PL Web portal includes metrics desired by the clinic for the tracking program	No action indicated
	Adaptability: Ability to adapt PL to unique clinic workflows	Ability to tailor PL, such as who receives PL messages Ability to adapt the web portal to show desired information	No action indicated
**Implementation process**			
	Engaging key stakeholders: Function and roles of the clinic team	Leadership at the clinic (CEO and clinic supervisor) is committed to the program Teamwork within the site to identify clients likely to benefit from PL and prioritize their enrollment Evolution of roles over time, that is, the supervisor has more responsibility during the approval phase and then responsibility transfers to coordinators	Consider creating an opportunity for coordinators at different sites to interact with each other and share their experiences in order to build engagement as a community of practice
	Planning: Planning	Teams feel better prepared for PL implementation when having a plan of who to enroll first, who will manage PL, and how PL will fit into their workflow Proactive engagement with information technology security, anticipating the need for key approvals and proactively seeking them Soft launch with trial run, including mock patients and messages, to get clinic staff engaged and comfortable Create plans with milestones and timelines	Plan for clinician and other clinic staff engagement by asking for their input for program improvement
	Engaging innovation participants: Initial success of rollout	Early success during implementation (clinicians buy-in; clients loving it)	Emphasize individual-level features first (check-ins, resources) and phase in the community board when there are enough participants to make it engaging
**Characteristics of individuals**			
	Knowledge and beliefs about the innovation: Perceptions of PL	Positive attitudes toward PL and its implementation by the PL coordinator, providers, and other staff	No action indicated

^a^CFIR: Consolidated Framework for Implementation Research.

^b^PL: PositiveLinks.

### Barriers

Six CFIR constructs were associated with eight common barriers. A summary of those barriers and their recommended action items are shown in Table 3. Barriers associated with PL *compatibility* were common (n=3) and related to mHealth technology either directly or indirectly. Specific examples expressed by the respondents included not being able to get Wi-Fi in the clinic to download PL on their phones and the release of laboratory results on PL prior to review by the medical provider, which was in opposition to some clinic’s usual workflows.

The remaining barriers were associated with different CFIR constructs. *Policy and incentives* represented a single barrier related to privacy concerns, both from clients and administrators concerned about regulatory compliance. Issues in implementation *planning* arose in relation to obtaining institutional approvals for PL use, in part because of unclear internal review processes. Barriers associated with *available resources* focused on phone availability and staffing effort to manage the PL program. PL’s *adaptability* by clinics created potential barriers to uptake, with suggestions for allowing tailoring of the PL platform appearance to appeal to unique clinic populations. To address these barriers, the team developed the following action items: (1) create a document that helps sites anticipate potential IT challenges with tips on how to address in advance and (2) provide examples from other sites, including stories or case studies related to how sites addressed common problems and how long their implementation process took.

**Table 3:** Barriers to PositiveLinks implementation.

CFIR^a^ domain and construct: barrier		Example	Action item
**Outer setting**			
	External policy and incentives: Privacy	Privacy concerns from information privacy officers Clients are concerned about privacy issues	Create a document with suggestions to help sites prepare for anticipated challenges. Include tips such as figuring out who key decision makers are, what permissions are needed, and identifying all the people the team will likely need on board (eg, privacy, security, and clinical)
**Inner setting**			
	Compatibility: Information technology	Wi-Fi access at enrollment locations Phone related (permission to trust app, troubleshooting phone technology) PL not integrated with EMRb	PL^c^ prioritizes EMR integration
	Compatibility: Mismatch of goals/priorities	Clinicians focused on benefits to patients; may not be aware of PL goals set by an external decision maker Mismatch between desire of the clinic director to implement PL and the buy-in from staff carrying out the implementation	Develop new strategies for communicating among site stakeholders about goals and priorities
	Compatibility: Clinic workflow/structure	Concern about patients seeing their lab results in PL before their appointment Competing priorities for clinic staff	Allow sites to tailor the lab feed to meet their own needs; consider only releasing lab values after provider review Emphasize that clinics consider multiple responsibilities of staff and discuss ways to fit PL into existing workflow based on their unique processes
	Available resources: Resources	Acquiring cell phones and coordinating cell phone payments Limited resources to handle enrollments, manual entry of lab results, and appointments in PL Staffing numbers and capacity to successfully enact an mHealth intervention	Continue sharing the reference document outlining the different phone service providers and how to pay them Create a learning module specific to cell phone payments; provide a customizable template for sites Re-emphasize staffing needs, including that needs may change over time
**Implementation process**			
	Planning: Preimplementation approvals	Unclear how to initiate internal approval processes Decisions to adopt PL disconnected from PL users Initial concern over the mechanics and length of time needed to implement PL	Provide examples from other sites Consider writing stories/case studies about implementation processes that have succeeded at other sites, including how long it takes to go through each step of the process
	Engaging key stakeholders: Buy-in	Doctors see PL as “another thing to do” Coordinator unsure of the level of buy-in from all staff including nurses Coordinators need targeted engagement strategies to keep clients and providers using the app	Consider providing literature on provider engagement Remind coordinators that PL can still be beneficial to patients if providers do not engage in the app Clearly articulate that “providers” can include other staff roles, not only doctors Consider retraining providers if there is uneven engagement or high turnover Consider involving the “frontline” staff in early implementation decision making and planning to improve engagement, motivation, and compatibility Set realistic goals for PL participation; provide guidance on what goals to aim for and how to track goals
**Innovation characteristics**			
	Adaptability: Adaptability and design of PL	PL is developed externally by a site that is different than the expansion site Some PL features do not meet the preferences of clients (older patients may have difficulty or lack of interest in a mobile app; younger patients may prefer a more upgraded interface)	Anticipate needing to adapt PL from one clinic population to another; seek more input from the clinic staff up front about their clients’ needs Ensure that app updates and upgrades continue on an ongoing basis following feedback from users

^a^CFIR: Consolidated Framework for Implementation Research.

^b^EMR: emergency medical record.

^c^PL: PositiveLinks.

## Discussion

### Principal Findings

This work demonstrates the feasibility of applying the CFIR to the evaluation of mHealth implementations. Rapid evaluation methods using a determinants framework were deemed ideal for interim analysis, simultaneously providing for rigorous assessment of PL implementation processes, identification of specific barriers and facilitators of implementation, and timely refinement of our implementation support program [14].

One of the goals of this analysis was to determine if CFIR could adequately capture the important factors in mHealth implementation. We found that the constructs within CFIR are sufficient to pick up mHealth-specific considerations that may impact successful implementation. Although most content captured within our functional *technology* code was also coded with a CFIR construct, its inclusion in our final codebook enabled more rapid data reduction and analysis to identify instances of delayed or stalled PL uptake due to the clinic’s or health system’s technology-related barriers. Technology played a role in each of the CFIR domains. The addition of a functional code for *technology* allowed the evaluation team to extract segments of interviews specific to technology aspects of the implementation support program. This enabled us to target our rapid analysis; however, CFIR alone is suitable to cover the concepts arising in mHealth intervention-related interviews.

Preliminary results from the first four PL expansion sites identified *compatibility*, e*ngaging key stakeholders*, and *innovation participants and planning* as important CFIR constructs associated with early PL implementation. The primary barriers identified in our early results were related to technology and mHealth. mHealth interventions require support from a broad range of stakeholders, including clinic leadership and administration, clinic staff, security and privacy officers, and IT personnel. One recommendation that emerges from this finding is that sites intending to implement an mHealth intervention should engage their security and technology leaders and staff early in the process to streamline implementation and help to avoid or minimize technology-related barriers, such as multiple levels of review and permissions that can be required by large systems. Further, mHealth interventions are unique in their need for continuous updates following their initial implementation. Unlike more discrete interventions, mHealth interventions are not complete once they are implemented but instead require ongoing technological maintenance and support. We identified a useful application of the CFIR for identifying barriers and facilitators at PL sites while PL implementation processes were underway. This enabled us to provide specific action items for the PL implementation team, and it resulted in iterative refinement of our PL implementation strategy. The evaluation was successful in providing interim CFIR-informed feedback to implementation stakeholders rather than waiting for the end of implementation to assess its success. This model could be useful in other implementations that occur on a rolling or ongoing basis.

The results of our evaluation led to revisions in a detailed implementation manual that is provided to new expansion sites. This manual includes information about the PL program itself, including a detailed description of all app features and components of the web portal. Revisions to the implementation manual included (1) a description of important milestones in the implementation process including developing a budget and IT flows, (2) information about gathering necessary security and privacy approvals, and (3) phone logistics. This manual is essential to the successful implementation of PL at new sites and is continually updated based on feedback learned from the implementation interviews using the rapid feedback approach taken by the evaluation and implementation teams.

Rapid evaluation was critical to providing timely feedback to the implementation team and expansion sites. Because the implementation team is continuously implementing PL and working to meet the immediate needs of expansion sites, it is important to plan regular communication between the evaluation and implementation teams. The frequency of communication should be based on timing of interviews and the emergence of new sites. Both teams should establish an integrated flow that allows for two-way channels of communication from the implementation team to the evaluators regarding the status of pending sites, as well as from the evaluators to the implementers regarding recommended changes to the implementation process.

The results of this rapid interim evaluation suggest that health systems looking to adopt new mHealth apps to improve patient engagement and outcomes will need to consider adopting a streamlined approach to decision making and IT infrastructure, including security, to have successful mHealth implementation. Our findings are consistent with other recent evaluations of mHealth interventions [33,34] that used CFIR to identify factors influencing implementation, including characteristics of the innovations themselves, as well as the local and institutional contexts in which they are being adopted. Scale-up of technological innovation is a challenging process and can be inhibited by organizational factors external to the intervention.

Future work will include incorporating interviews with patients who represent another important stakeholder group in the implementation of mHealth. Additional next steps will include incorporating the CFIR valence attribution as the number of sites increase and we become able to identify the positive and negative attributions of each implementation.

### Limitations

One of the main limitations of this evaluation was the lack of provider interviews. Our provider recruitment experience echoes others in the implementation literature, requiring perseverance of our PL evaluation team and considerably longer recruitment times than predicted [35]. Providers represent a key stakeholder group in mHealth chronic disease interventions, and their participation in the evaluation of such interventions is critical. For interventions like PL that are designed to improve connections between users with chronic medical conditions and members of their health care teams, intervention effectiveness is dependent on provider uptake. Engaging providers in evaluations of PL implementation is essential for the recognition of and response to the unique barriers to PL use.

### Conclusion

This study describes the use of the CFIR to guide iterative refinement of an implementation strategy to facilitate dissemination of our mHealth intervention. Our findings highlight the unique characteristics of mHealth interventions and the multilevel factors that must be considered when planning for their implementation in health care settings. The flexibility and comprehensiveness of CFIR appear to be sufficient to capture concepts within the interviews that we conducted and likely would be applicable to the evaluation of other mHealth interventions. Strategies for rapid evaluation may be particularly important in the realm of mHealth, where the field can move quickly. Rapid evaluation methods that are rigorous and responsive to the experiences of early mHealth adopters can better inform best practices for mHealth implementation. Increasing provider feedback will also enable more impactful and utility-focused evaluation.

## Abbreviations

CFIR: Consolidated Framework for Implementation Research
IT: information technology
mHealth: mobile health
PL: PositiveLinks
